# Synergy between Common γ Chain Family Cytokines and IL-18 Potentiates Innate and Adaptive Pathways of NK Cell Activation

**DOI:** 10.3389/fimmu.2016.00101

**Published:** 2016-03-22

**Authors:** Carolyn M. Nielsen, Asia-Sophia Wolf, Martin R. Goodier, Eleanor M. Riley

**Affiliations:** ^1^Department of Immunology and Infection, Faculty of Infectious and Tropical Diseases, London School of Hygiene and Tropical Medicine, London, UK

**Keywords:** CD25, antibody, IL-2, IL-15, synergy

## Abstract

Studies to develop cell-based therapies for cancer and other diseases have consistently shown that purified human natural killer (NK) cells secrete cytokines and kill target cells after *in vitro* culture with high concentrations of cytokines. However, these assays poorly reflect the conditions that are likely to prevail *in vivo* in the early stages of an infection and have been carried out in a wide variety of experimental systems, which has led to contradictions within the literature. We have conducted a detailed kinetic and dose–response analysis of human NK cell responses to low concentrations of IL-12, IL-15, IL-18, IL-21, and IFN-α, alone and in combination, and their potential to synergize with IL-2. We find that very low concentrations of both innate and adaptive common γ chain cytokines synergize with equally low concentrations of IL-18 to drive rapid and potent NK cell CD25 and IFN-γ expression; IL-18 and IL-2 reciprocally sustain CD25 and IL-18Rα expression in a positive feedback loop; and IL-18 synergizes with FcγRIII (CD16) signaling to augment antibody-dependent cellular cytotoxicity. These data indicate that NK cells can be rapidly activated by very low doses of innate cytokines and that the common γ chain cytokines have overlapping but distinct functions in combination with IL-18. Importantly, synergy between multiple signaling pathways leading to rapid NK cell activation at very low cytokine concentrations has been overlooked in prior studies focusing on single cytokines or simple combinations. Moreover, although the precise common γ chain cytokines available during primary and secondary infections may differ, their synergy with both IL-18 and antigen–antibody immune complexes underscores their contribution to NK cell activation during innate and adaptive responses. IL-18 signaling potentiates NK cell effector function during innate and adaptive immune responses by synergy with IL-2, IL-15, and IL-21 and immune complexes.

## Introduction

Natural killer (NK) cells are classically regarded as innate lymphocytes that, by cytotoxicity and cytokine production, are among the earliest responders to damaged, transformed, or infected host cells. In the context of infection, they may directly lyse infected cells that no longer express self MHC Class I, mediate antibody-dependent cellular cytotoxicity (ADCC) of opsonized cells, or secrete pro-inflammatory cytokines, such as IFN-γ, to activate phagocytes. During primary infection in a naive host, NK cells are activated by cytokines and contact-dependent signals from antigen-presenting cells [reviewed in Ref. (1)]; during secondary infection in a primed host, these innate signals are augmented by IL-2 from antigen-specific T helper cells (2–6) and by IgG immune complexes cross-linking FcRγIII (CD16) on the NK cell surface (7).

The precise cocktails of innate cytokines that most efficiently activate NK cells in response to any given pathogen, and how these cytokines synergize with adaptive responses, such as IL-2 and antibody to optimize the initial response to infection, are poorly described. Although numerous studies have reported NK cell activation by specific cytokines, they have tended to use very high concentrations of individual cytokines or combinations of cytokines, at a single concentration, to activate isolated populations of NK cells over rather long periods of time. For example, Aste-Amezaga et al. (8) demonstrated synergy between 1 ng/ml IL-12 and 100 IU/ml IL-2 for IFN-γ secretion from purified, B cell lymphoma-activated NK cells; Fehniger et al. (9) found that IL-12, IL-15, or IL-18 alone did not induce IFN-γ but that IFN-γ was produced by positively selected human NK cells after 24 h stimulation with 10 U/ml (approximately 2 ng/ml) IL-12 in combination with either 100 ng/ml IL-18 or 100 ng/ml IL-15; Son et al. (10) observed proliferation, CD25 upregulation, IFN-γ production, and cytotoxic activity in purified NK cells cultured for three days with 6 IU/ml IL-2 plus 1000 ng/ml IL-18; Trotta et al. (11) observed IFN-γ secretion from positively selected NK cells after 24 h stimulation with 10 ng/ml IL-12 plus either 100 ng/ml IL-18 or 10 μg/ml anti-CD16; and Leong et al. (12) described upregulation of CD25 (and subsequent enhanced responsiveness to IL-2) among purified NK cells cultured for 16 h with 50 ng/ml IL-18 plus either 100 ng/ml IL-15 or 10 ng/ml IL-12.

Although these studies, and others (13–16), produce a consistent picture of purified NK cells being able to proliferate and respond functionally to high concentrations of cytokines over periods of 16–72 h and are valuable in the context of developing NK cell-based therapies for cancer and other diseases, they provide little insight into the very early contribution that NK cells may make during infection. Indeed, there are no published studies that have systematically evaluated the kinetics, dose–response, and synergistic interactions leading to rapid and potent NK cell responses under conditions that mimic events early in primary infection. Similarly, there are limited data on the interaction between innate cytokines and components of the adaptive immune response to predict how NK cells might respond during secondary and subsequent infections.

In this study, we have conducted a detailed dose–response analysis of human NK cell CD25 and IFN-γ responses to IL-12, IL-15, IL-18, IL-21, and IFN-α at 6 and 18 h, and looked for synergy between low concentrations of these cytokines and IL-2. We have also investigated the capacity of IL-18 to synergize with FcRγIII (CD16) signaling to drive degranulation (CD107a) responses, in addition to CD25 and IFN-γ upregulation. Importantly, our assays have been carried out on unselected NK cells within peripheral blood mononuclear cells, in order to avoid inadvertent activation of NK cells during the purification process and in order to better reflect the cellular environment in which early responses to infection take place *in vivo*. Our data reveal potent and rapid activation of NK cells at cytokine concentrations that are orders of magnitude lower (and thus more physiologically relevant) than previously described. We also observe synergy between members of the common γ chain (γ_c_) cytokine family, which includes IL-15, IL-2, and to a lesser extent IL-21, and IL-18, at exceedingly low concentrations; synergy between IL-18 and FcRγIII signaling in initiation of ADCC reactions; and synergistic, reciprocal upregulation of IL-18 receptor (IL-18Rα) and the high affinity IL-2 receptor (CD25) by IL-18 and IL-2 in an effective positive feedback loop.

In summary, these data indicate that NK cells can be very rapidly activated by exceedingly low doses of innate cytokines, such as might be found within minutes or hours at the site of infection, and can be further potentiated by adaptive immune responses (T cell IL-2 and antigen–antibody immune complexes) that are expected to occur upon re-infection.

## Materials and Methods

### Study Subjects

Volunteers were recruited from among staff and students at the London School of Hygiene and Tropical Medicine. All subjects gave written consent under a protocol for recruitment of blood donors approved by the LSHTM ethics committee (reference # 5520) to provide ≤50 ml venous blood.

### PBMC Preparation and Culture

PBMCs were isolated from heparinized venous blood on a Ficoll–Hypaque gradient and cryopreserved in liquid nitrogen. Before use, PBMCs were thawed into complete medium [RPMI 1640, supplemented with 100 U/ml penicillin/streptomycin and 20 mM l-glutamine (Life Technologies) and pooled 10% human AB plasma], washed, and rested for a minimum of 30 min before use.

PBMCs (2 × 10^5^/well) were cultured for 6 or 18 h in 5% CO_2_ at 37°C at in 96-well *U*-bottom plates (Nunc) in complete medium with or without varying concentrations and combinations of recombinant human IL-2, IL-12, IL-15, IFN-α, IL-21 (all from PeproTech), IL-18 (R&D Biosystems), or inactivated influenza virus H3N2 (1 μg/ml, NIBSC). GolgiStop (containing Monensin, 1/1500 concentration, BD Biosciences) and GolgiPlug (containing brefeldin A, 1/1000 final concentration, BD Biosciences) were added after 3 and 15 h (in 6 and 18 h cultures, respectively) in experiments where intracellular IFN-γ was a read-out. Similarly, anti-CD107a antibody (A488-conjugated, BD Biosciences) was included in the medium for the entirety of cell culture when CD107a upregulation was a read-out.

For activation *via* CD16 cross-linking, 96-well flat-bottom plates (Nunc) were coated with anti-human CD16 (BD Biosciences) or an isotype-matched control antibody (mIgG1κ, BD Biosciences) overnight at 4°C. Plates were washed with sterile PBS before addition of 4 × 10^5^ PBMC per well. Cells were harvested after 6 or 18 h. GolgiStop, GolgiPlug, and anti-CD107a were used, as described above.

### Flow Cytometry

PBMCs were stained in 96-well *U*-bottom plates, as described previously (6). Briefly, cells were stained with fluorophore-labeled antibodies to cell surface markers then fixed, permeabilized (Cytofix/Cytoperm, BD Biosciences), and stained for intracellular molecules. The following monoclonal antibodies were used: anti-CD3-V500, anti-CD57-e450, anti-CD56-PECy7, anti-CD107a-A488, anti-IFN-γ-allophycocyanin, anti-CD16-allophycocyanin-H7, anti-CD16-allophycocyanin, anti-CD25-PerCPCy5.5, anti-IL-18Rα-PE, and anti-IL-18Rα-FITC (all BD or e-Biosciences). Anti-IL-12Rβ2 (R&D Systems) was conjugated to PerCP/Cy5.5 in-house (EasyLink PerCP/Cy5.5^®^ Abcam). Cells were acquired on an LSRII flow cytometer (BD Biosciences) using FACSDiva software. Data analysis was performed using FlowJo v10 (Tree Star). Gating strategies are detailed in figure legends, with gates set on unstimulated cells (medium alone or isotype controls, with no cytokine stimulation) and applied in a standard format.

### Statistical Analyses

Statistical analysis of flow cytometry data was performed using Prism 6 (GraphPad), as specified in figure legends. Data were excluded when the gated cell subset contained fewer than 100 cells. Paired Wilcoxon signed-rank tests were used to compare responses between stimulation conditions and ANOVA tests for linear trend were used to analyze cytokine titrations. Formal tests for synergy using regression analysis with an interaction term, and linear regression adjusting for confounding factors were performed in STATA (V.14.0). All statistical tests are two-sided ****p ≤ 0.0001, ***p < 0.001, **p < 0.01, *p < 0.05. Sample sizes are stated in figure legends.

## Results

### Common γ Chain Family Cytokines Synergize with IL-18 to Drive CD25 Expression on NK Cells

**Figure 1:**
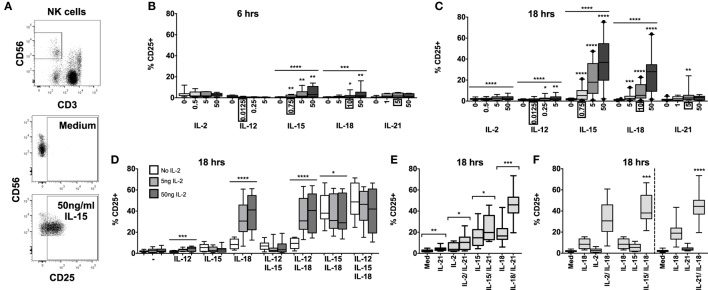
**IL-18 and IL-15 both independently drive CD25 but interact differently with IL-2**. PBMCs were stimulated for 6 or 18 h *in vitro* and upregulation of NK cell surface expression of CD25 was measured in response to Med (medium alone), IL-2, IL-12, IL-15, IL-18, or IL-21. Representative flow cytometry plots show gating of CD3^−^CD56^+^ NK cells and surface expression of CD25 on unstimulated and IL-15-stimulated NK cells (50 ng/ml) **(A)**. CD25 expression on NK cells was measured after stimulation with Med, IL-2, IL-12, IL-15, IL-18, or IL-21 (concentrations in nanograms per milliliter as labeled) for 6 h **(B)** or 18 h **(C)** (*n* = 6–22, data from two to three experiments). Concentrations in boxes indicate those used in following graphs. CD25 expression on NK cells was also measured after stimulation with a titration of IL-2 (0, 5, and 50 ng/ml) in combination with IL-12 (12.5 pg/ml), IL-15 (0.75 ng/ml), and/or IL-18 (10 ng/ml) for 18 h **(D)** (*n* = 7–8, data from two experiments). CD25 expression on NK cells was also measured following stimulation with 5 ng/ml IL-21 in combination with 5 ng/ml IL-2, 0.75 ng/ml IL-15, or 10 ng/ml IL-18 after 18 h **(E)** (*n* = 8, data from one experiment). CD25 expression after stimulation for 18 h with a combination of IL-18 (10 ng/ml) and γ_c_ cytokines was summarized to facilitate comparison between IL-2 (5 ng/ml), IL-15 [0.75 ng/ml; both from **(D)**], and IL-21 [5 ng/ml; from **(E)**] **(F)** (*n* = 7–8, data from one to two experiments). Box plots show the 5th to 95th percentile range. Data were analyzed using paired Wilcoxon signed-rank tests [**(B,C,F)** no lines; lowest concentration compared to Med, **(E)** capped lines; IL-2/IL-18 and IL-15/IL-18 compared to IL-18 alone, (F)] or ANOVA tests for linear trend for trend analysis across increasing cytokine concentrations including Med [**(B–D)**, uncapped lines]. ****p < 0.0001, ***p < 0.001, **p < 0.01, *p < 0.05.

PBMCs were stimulated with increasing concentrations of IL-2, IL-12, IL-15, IL-18, or IL-21, and NK cell surface expression of CD25 was measured after 6 or 18 h (Figures 1A–C). Upregulation of CD25 is of interest as a marker of NK cell activation and, more specifically, increased sensitivity to IL-2; indeed, NK cell production of IFN-γ in response to picomolar levels of IL-2 has been shown to be CD25 dependent (12). The highest cytokine concentrations tested reflect those widely used as positive controls by ourselves (5, 6, 17) and others; cytokines were then titrated to concentrations at least fivefold lower than the lowest previously described effective concentration.

IL-15 and IL-18 each, independently, drive CD25 expression in a dose- and time-dependent manner. Significant CD25 expression could be detected within 6 h among cells cultured with cytokine concentrations as low as 0.75 ng/ml IL-15 and 10 ng/ml IL-18 (Figure 1B) but CD25 expression was markedly higher after 18 h for both cytokines and evident at the lowest cytokine concentrations tested (0.75 ng/ml IL-15 and 5 ng/ml IL-18) (Figure 1C). For IL-15, this is 6-fold lower than the previously described minimal concentration (14, 16) for upregulation of CD25, and 10- to 1000-fold lower than previously used concentrations of IL-18 (12, 15). Incubation of PBMC with IL-2, IL-12, and IL-21 induced minimal, albeit statistically significant, expression of CD25 on NK cells at 18 h, but not at 6 h.

To investigate potential synergies between cytokines in driving CD25 expression on NK cells, PBMCs were stimulated with combinations of IL-12, IL-15, and IL-18, with or without varying concentrations of IL-2, to model early NK cell activation in response to primary pathogen infection (innate cytokines only, no IL-2) and secondary infection (innate cytokines plus IL-2 from memory CD4^+^ T cells). We selected the lowest concentrations of IL-12 and IL-15 that had been tested singly (12.5 pg/ml and 0.75 ng/ml, respectively) and, for consistency with our own previously published work (5, 6, 17), we used the middle concentration of IL-18 (10 ng/ml). The middle concentration of IL-21 (5 ng/ml), an adaptive γ_c_ cytokine, was selected to permit later comparisons with IL-2.

Consistent with the data presented in Figure 1B, CD25 expression was very low after 6 h, and there was no significant evidence of synergism between cytokines (data not shown). However, after 18 h, the data clearly showed synergy between IL-18 and IL-2 in driving NK cell CD25 expression (trend analysis *p* < 0.0001 for IL-18 in combination with increasing concentrations of IL-2) with 5 ng/ml IL-2 in combination with 10 ng/ml IL-18 giving CD25 expression levels equivalent to those seen with 50 ng/ml IL-18 alone (Figures 1C,D). Although adding IL-12 to a cocktail of IL-2 plus IL-18 did not further enhance CD25 expression, including a low concentration of IL-12 (0.0125 ng/ml) in the cultures did permit detection of a modest IL-2 dose response (Figure 1D) and much higher, though less physiological, concentrations of IL-12 (1–10 ng/ml) do synergize with IL-18 to drive CD25 expression (Figure S1 in Supplementary Material) (12, 18). There was also strong evidence that IL-15 synergizes with IL-18 to enhance NK cell CD25 expression (Figure 1F, test for interaction IL-15 and IL-18, *p* = 0.009).

By contrast, IL-15-driven CD25 upregulation was partially inhibited by IL-2: there was a trend for the proportion of NK cells expressing CD25 to decrease with increasing concentrations of IL-2 in all cytokine combinations that included IL-15, with a statistically significant impact observed in NK cells stimulated with IL-15 plus IL-18 (median without IL-2 = 38.1% vs. median with high concentration IL-2 = 29.0%; linear test for trend, *p* = 0.04; Figure 1D).

In a separate set of experiments, we also tested IL-21 and IFN-α for their ability to synergize with IL-2, IL-15, and IL-18 to drive CD25 expression on NK cells. There was no evidence that IFN-α alone induced CD25 expression nor did it enhance CD25 expression in combination with other cytokines (Figure S2 in Supplementary Material). However, IL-21 in combination with IL-2, IL-15, or, in particular, IL-18 significantly enhanced CD25 expression compared to these cytokines alone (Figure 1E). Indeed, there was clear evidence of synergy between IL-21 and IL-18 driving CD25 expression (Figure 1F, test for interaction IL-21 and IL-18, *p* < 0.0001).

In summary, these data indicate that at least three different cytokines that signal *via* the common γ chain (CD132) can individually synergize with the IL-18 pathway leading to rapid upregulation of CD25 expression on NK cells, and at much lower cytokine concentrations than previously appreciated (Figure 1F). As IL-15 and IL-18 are produced primarily by dendritic cells, monocytes, and macrophages, and as IL-2 and IL-21 are primarily T cell-derived, these combinations of cytokines allow for very early NK cell activation – when cytokine concentrations are still extremely low – *via* both innate and adaptive immune pathways. Moreover, there is evidence of homeostatic regulation of NK cell activation *via* γ_c_ cytokines, as illustrated by inhibition of IL-15-driven CD25 upregulation by IL-2.

### Common γ Chain Cytokines Synergize with IL-18 to Drive Rapid and Extensive IFN-γ Production by NK Cells

**Figure 2:**
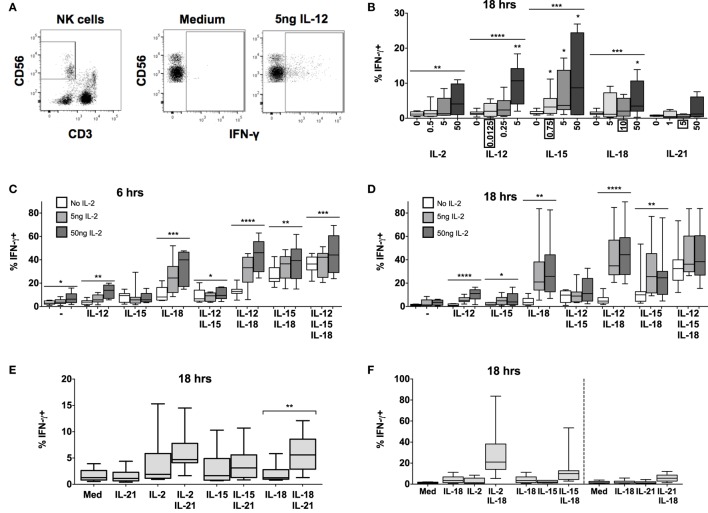
**IL-15 and IL-18 can synergize to drive IFN-γ in absence of IL-12 or IL-2**. PBMCs were stimulated for 6 or 18 h *in vitro* and production of intracellular IFN-γ by NK cells was measured in response to Med (medium alone), IL-2, IL-12, IL-15, IL-18, or IL-21. Representative flow cytometry plots show gating of CD3^−^CD56^+^ NK cells and percentage of CD56^+^ cells that are positive for intracellular IFN-γ on unstimulated and IL-12-stimulated NK cells (5 ng/ml) **(A)**. IFN-γ production by NK cells was measured after stimulation with Med, IL-2, IL-12, IL-15, IL-18, or IL-21 (concentrations in nanograms per milliliter as labeled) for 18 h **(B)** (*n* = 4–9, data from one to three experiments). Concentrations in boxes indicate those used in following graphs. IFN-γ production by NK cells was also measured after stimulation with a titration of IL-2 (0, 5, and 50 ng/ml) in combination with IL-12 (12.5 pg/ml), IL-15 (0.75 ng/ml), and/or IL-18 (10 ng/ml) for 6 h **(C)** or 18 h **(D)** (*n* = 7–8, data from two experiments). IFN-γ production by NK cells was also measured following stimulation with 5 ng/ml IL-21 in combination with 5 ng/ml IL-2, 0.75 ng/ml IL-15, or 10 ng/ml IL-18 after 18 h **(E)** (*n* = 8, data from one experiment). IFN-γ expression after stimulation for 18 h with a combination of IL-18 (10 ng/ml) and γ_c_ cytokines was re-plotted to facilitate comparison between IL-2 (5 ng/ml), IL-15 [0.75 ng/ml; both from **(D)**], and IL-21 [5 ng/ml; from **(E)**] for 18 h **(F)** (*n* = 7–8, data from one to two experiments). Box plots show the 5th to 95th percentile range. Data were analyzed using paired Wilcoxon signed-rank tests [**(B)**: no lines; lowest concentration compared to Med; **(E)** capped lines] or ANOVA tests for linear trend for trend analysis across increasing cytokine concentrations including Med [**(B–D)**, uncapped lines]. ****p < 0.0001, ***p < 0.001, **p < 0.01, *p < 0.05. *n* ≥ 8, other than for IL-21 titration **(B)** where *n* = 4.

Upregulation of CD25 primes NK cells for enhanced subsequent responses to IL-2 (12) but is not, in itself, a read-out of NK cell effector function. We have therefore characterized the effect of combining low concentrations of different cytokines on IFN-γ production, assessed by intracellular staining after incubation of PBMC with increasing concentrations of individual cytokines or cytokine combinations (Figure 2).

Increasing concentrations of IL-2, IL-12, IL-15, or IL-18 (but not IL-21) each, individually, induced significant IFN-γ production by NK cells at 18 h, although the proportions of IFN-γ^+^ cells rarely exceeded 10% even at the highest cytokine concentrations (Figure 2B). However, combining IL-18 (at a concentration of 10 ng/ml), with as little as 5 ng/ml IL-2 (which alone did not drive IFN-γ) not only induced IFN-γ in much higher proportions of NK cells (>20%) but did so within 6 h of incubation (Figure 2C). Although this interaction appears additive at 6 h (test for interaction, *p* = 0.22) by 18 h the interaction is highly synergistic (test for interaction, *p* = 0.006), possibly as a result of IL-18 induced upregulation of the high affinity IL-2R.

In contrast to what we observed for CD25 expression, there was no evidence of antagonism or competition between γ_c_ cytokines in their induction of NK cell IFN-γ. On the contrary, there was evidence of additive or synergistic interactions between γ_c_ cytokines with increasing concentrations of IL-2 modestly but significantly enhancing NK cell IFN-γ responses to IL-15 + IL-18 (Figures 2C,D). Low concentrations of IL-15 (Figures 2C,D) and IL-21 (Figure 2E) also enhanced IL-18-induced NK cell IFN-γ production, but to a lesser extent than IL-2 (Figures 2C,D,F). Although IL-15 plus IL-18 has previously been shown to enhance NK cell IFN-γ, as measured by ELISA, the effects described here were apparent at an IL-15 concentration (0.75 ng/ml) markedly lower than previously described (5 ng/ml) (14).

Again, as for CD25 expression, we found no evidence of a role for IFN-α in NK cell IFN-γ production (Figure S2 in Supplementary Material); this is in contrast to published data (19). Low concentrations of IL-12 (0.0125 ng/ml) – alone or in combination with IL-2 or IL-21 – had minimal effects on IFN-γ production (Figures 2C–E), but did enhance IFN-γ production in combination with IL-15 and IL-18 at later time points. High concentrations of IL-12 (≥1 ng/ml) synergized strongly with IL-18 to drive both IFN-γ and CD25, although we suggest that these do not reflect physiological conditions (Figure S1 in Supplementary Material) (9, 11, 12, 15, 20). Overall, however, as little as 5 ng/ml IL-2 in combination with low concentrations of IL-18 (10 ng/ml) and IL-12 (12.5 pg/ml) was the optimal combination for NK cell IFN-γ induction at 18 h.

In summary, therefore, γ_c_ cytokines (IL-2, IL-15, and IL-21) in combination with IL-18 induce very rapid and extensive IFN-γ production by NK cells (Figure 2F). Although IL-2 seems to be the most potent of these, at least at the cytokine concentrations tested, the ability of IL-15 to augment IFN-γ production offers a route for rapid, innate activation of NK cells prior to the differentiation of IL-2 secreting T cells. Of interest, given the very large body of work describing IFN-γ induction by combinations of IL-12 and IL-18, γ_c_ cytokines synergize with IL-18 at extremely low concentrations. It is possible therefore that, *in vivo*, IL-12 may contribute to NK cell IFN-γ production when γ_c_ cytokines are lacking, such as during primary exposure (when IL-2 from antigen-specific T cells may be limiting) or later in infection when IL-15 signaling is reduced by changes in receptor expression (21, 22).

### IL-18 Signaling Sustains IL-18Rα Expression on NK Cells

**Figure 3:**
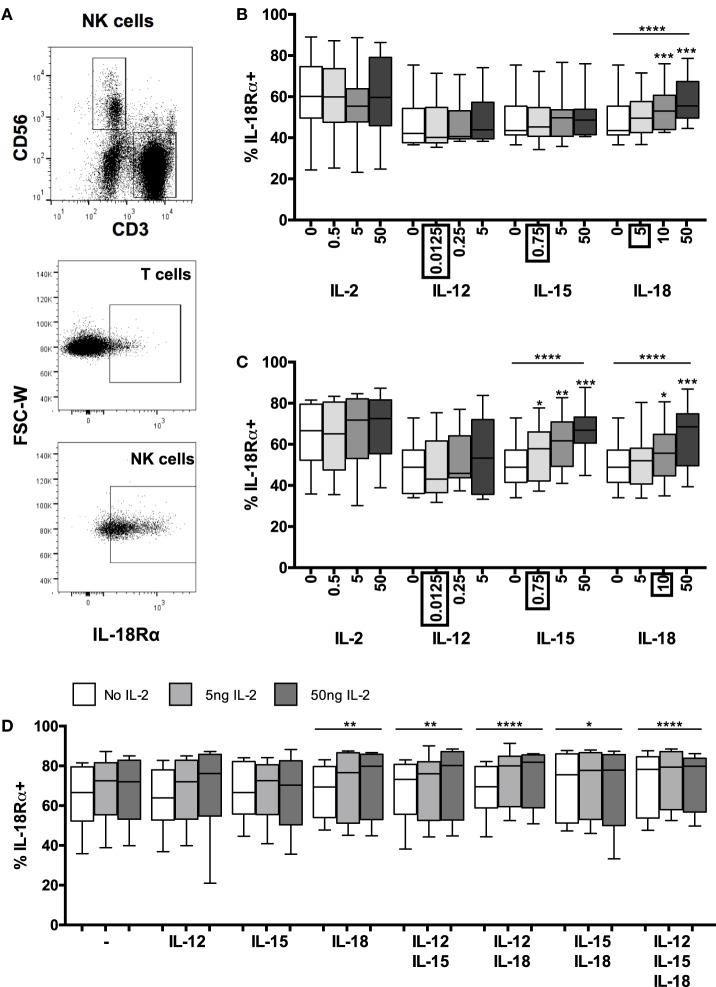
**Positive feedback from IL-18 induces IL-18R**. PBMCs were stimulated for 6 or 18 h *in vitro* and changes in NK cell surface expression of IL-18Rα was measured in response to Med (medium alone), IL-2, IL-12, IL-15, IL-18, or IL-21. Representative flow cytometry plots show gating of CD3^+^ T cells, CD3^−^CD56^+^ NK cells, and surface expression of IL-18Rα on unstimulated T cells and NK cells for IL-18Rα-FITC [N.B. as used in **(D)**; IL-18Rα-PE used in **(B,C)**] **(A)**. IL-18Rα expression on NK cells was measured after stimulation with Med, IL-2, IL-12, IL-15, IL-18, or IL-21 (concentrations per milliliter as labeled) for 6 **(B)** or 18 h **(C)** (*n* = 7–11 data from one to two experiments). Concentrations in boxes indicate those used in following graphs. IL-18Rα expression on NK cells was also measured after stimulation with a titration of IL-2 (0, 5, and 50 ng/ml) in combination with IL-12 (12.5 pg/ml), IL-15 (0.75 ng/ml), and/or IL-18 (10 ng/ml) after 18 h **(D)** (*n* = 8, data from two experiments). Box plots show the 5th to 95th percentile range. Data were analyzed using paired Wilcoxon signed-rank tests [**(B–D)** no lines; compared to Med] or ANOVA tests for linear trend for trend analysis across increasing cytokine concentrations including Med [**(B–D)**, uncapped lines]. ****p < 0.0001, ***p < 0.001, **p < 0.01, *p < 0.05.

As IL-18 alone is able to induce both CD25 and IFN-γ expression within 6 h (Figures 1B and 2C), we hypothesized that maintaining the capacity for IL-18 signaling might be required for optimal NK cell activation. Thus, the sustained or enhanced expression of the IL-18R may contribute to the synergy between IL-18 and γ_c_ cytokines. To determine whether, and if so which, cytokines regulate IL-18R expression, NK cell surface expression of IL-18Rα [CD218a, the receptor component required for signaling (23)] was measured after 6 or 18 h of PBMC culture with IL-2, IL-12, IL-15, IL-18, IL-21, or IFN-α, alone and in combination (Figure 3; Figure S2 in Supplementary Material). It was immediately obvious that resting levels of IL-18Rα expression are extremely variable between donors with the proportion of resting NK cells expressing the receptor varying from approximately 20% to >80% (Figures 3B,C). Polymorphisms affecting DNA methylation within the promoter region of *IL18R1*, the gene encoding IL-18Rα, and subsequent transcription of the gene have been reported and may in part explain this variation (24, 25) and we have previously observed lower levels of IL-18Rα expression in human cytomegalovirus (HCMV)-infected individuals than in HCMV-uninfected individuals (6). Despite this inter-individual variation, resting levels of IL-18Rα expression are very high in comparison to resting levels of the high affinity IL-2R (as defined by expression of the IL-2Rα chain, CD25; see Figures 1B,C) and fully functional IL-12R (as defined by expression of IL-12R-β2; see Figure S2 in Supplementary Material) and may explain the very rapid (within 6 h) NK cell response to exogenous IL-18 (Figures 1B and 2C). Indeed, we observed a weak but statistically significant correlation between resting levels of NK cell IL-18Rα expression and upregulation of CD25 following IL-18 stimulation [*n* = 18, 50 ng/ml IL-18, linear regression (adjusting for use of PE- and FITC-conjugated anti-IL-18Rα) *R*^2^ = 0.241, *p* = 0.046].

Contrary to previously published data indicating that IFN-α (13) and IL-12 (9, 11, 13, 20) can individually induce IL-18R mRNA (9, 13), IL-18Rα protein expression (11), or IL-18R expression (20) in human NK cells, and that IL-12/STAT4 signaling induces IL-18Rα expression in mice (26, 27), we found no increase in surface expression of IL-18Rα in response to increasing concentrations of either IFN-α (Figure S2 in Supplementary Material) or IL-12 (Figure 3B) with or without IL-2 (Figure 3C). There are several likely explanations for this discrepancy. For example, in previous studies, exogenous IL-2 was routinely added to NK cell cultures and IFN-α was used at much higher concentrations (13); NK cells were stimulated with very high concentrations of IL-12 (9, 11); IL-18R mRNA was assessed rather than IL-18R protein (9, 13); NK cells were purified by positive selection which may, in itself, contribute to subsequent activation (9, 11); or components of the IL-18R other than IL-18Rα were measured (9, 13, 20). Nevertheless, our data suggest that at physiological concentrations, IL-2, IL-12, and IFN-α have little, if any, effect on IL-18Rα expression. We also observed no effect of IL-21 on IL-18Rα expression (Figure S2 in Supplementary Material).

By contrast, we found clear evidence of concentration-dependent upregulation of NK cell IL-18Rα expression in response to IL-15 alone and IL-18 alone (Figures 3B,C). Ten nanograms per milliliter of IL-18 were sufficient to upregulate IL-18Rα within 6 h (Figure 3B) (*p* = 0.0010) and this effect was sustained at 18 h (Figure 3C) (*p* = 0.0003). IL-15 had no effect on IL-18Rα expression at 6 h, but as little as 0.75 ng/ml IL-15 was sufficient to upregulate IL-18Rα expression by 18 h (Figures 3B,C) (*p* = 0.019). Overall, IL-15 and IL-18 each increased the proportion of NK cells expressing IL-18Rα by approximately 15% (median percentage of IL-18Rα^+^ NK cells: 50.1% medium only; 64.6% with 50 ng IL-18; 66.8% with 50 ng IL-15). The ability of IL-18 to rapidly augment expression of its own receptor is suggestive of a positive feedback loop, allowing for enhanced IL-18 signaling, continued synergism with other signaling pathways and efficient induction of NK cell effector functions in the first few hours of infection.

We next considered whether other cytokines might synergize with IL-18 to further enhance IL-18Rα expression. Increasing concentrations of IL-2, either alone or in combination with IL-12 or IL-15 had no significant effect on IL-18Rα expression at either 6 h (not shown) or 18 h (Figure 3D). However, IL-2 modestly but significantly enhanced the effects of IL-18 in a dose-dependent manner, and there was an additive effect of combining IL-15 and IL-18 in the absence of IL-2 (Figure 3D). Addition of other cytokines to the IL-18 + IL-2 cocktail did not further enhance IL-18Rα expression. Although at the low cytokine concentrations used in these experiments (0.0125 ng/ml IL-12, 10 ng/ml IL-18) we saw no additive or synergistic effect of adding IL-12 to IL-18, at much higher concentrations (5 ng/ml IL-12 and 50 ng/ml IL-18) we did observe a significant (*p* < 0.0001) additive effect of these two cytokines increasing IL-18Rα expression (data not shown). These data support our contention that IL-18 is the key cytokine in initiating and sustaining NK cell responses under physiologically relevant conditions such as very early infection, and that NK cell responses that can be induced with very high (non-physiological) cytokine concentrations *in vitro* may not be relevant *in vivo*.

In summary, therefore, low concentrations of IL-18 rapidly and significantly upregulate the IL-18Rα subunit and this effect is augmented by low concentrations of IL-15 and (more substantially) by IL-2. Given that IL-18 alone is sufficient to induce expression of the high affinity IL-2R (Figures 1B,C), it seems that IL-18 and IL-2 synergistically and reciprocally upregulate their own and each other’s receptors in a potent positive feedback loop. The minimal role of low concentrations of IL-12 in this process may explain the limited synergies of exogenous IL-12 in the early NK cell IFN-γ response (Figure 2).

For completeness, we also examined expression of IL-12Rβ2 in response to single cytokines or cytokine combinations (Figure S3 in Supplementary Material). The proportion of NK cells expressing IL-12Rβ2 was transiently (seen at 6 h but not at 18 h) and very modestly enhanced by 10–50 ng/ml IL-18 and, in a slightly more sustained fashion, by 50 ng/ml IL-15, but the biological relevance of such small effects is unclear. There was no effect of exogenous cytokines on IL-12Rβ2 expression at the level of individual cells (as measured by MFI).

### IL-18 Synergizes with FcγRIII (CD16) Signaling to Augment NK Cell-Mediated Antibody-Dependent Cellular Cytotoxicity

**Figure 4:**
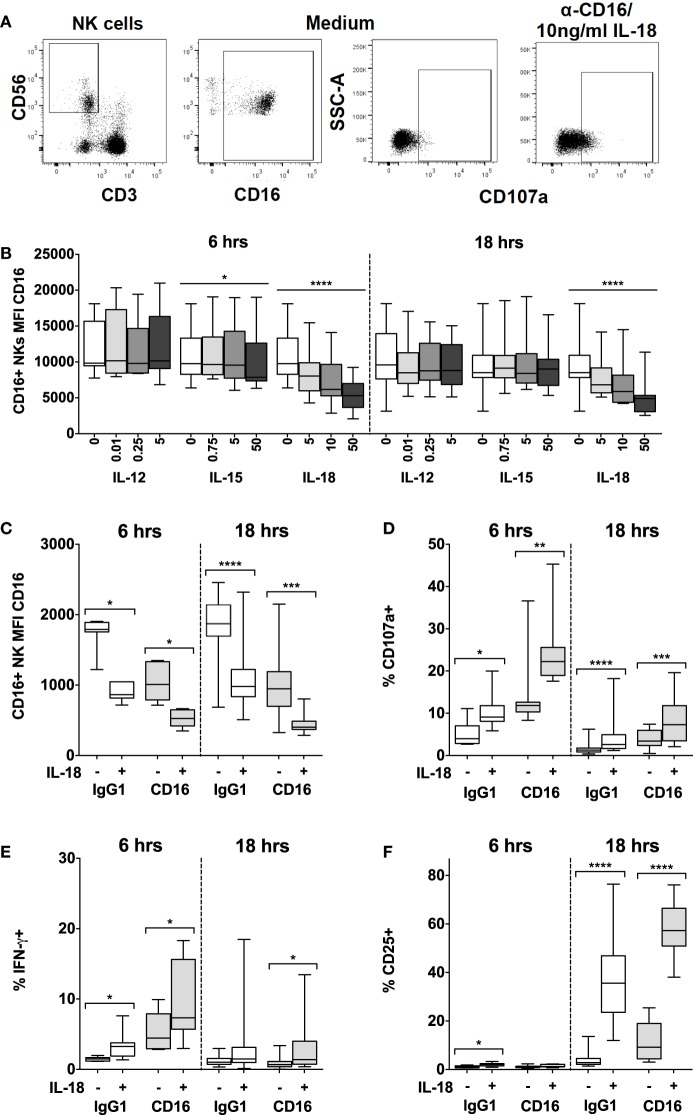
**IL-18 enhances responses to CD16 cross-linking, while simultaneously driving CD16 downregulation**. PBMCs were stimulated for 6 or 18 h *in vitro* and changes in CD16 mean fluorescence intensity (MFI) of CD16^+^ NK cells were measured in response to Med (medium alone), IL-12, IL-15, or IL-18. Representative flow cytometry plots show gating of CD3^−^CD56^+^ NK cells, and surface expression of CD107a or CD16 on unstimulated cells, or CD107a on NK cells activated with CD16 cross-linking and 10 ng/ml IL-18 **(A)**. CD16 MFI on CD56^dim^CD16^+^ NK cells was measured after stimulation with Med, IL-12, IL-15, or IL-18 (concentrations in nanograms per milliliter as labeled) for 6 or 18 h **(B)** (*n* = 9–13, data from two to three experiments). For the cross-linking assays (as described in the Section “Materials and Methods”), PBMCs were stimulated for 6 or 18 h *in vitro* with Med (medium alone), α-CD16 or its IgG1 isotype control, with (+) or without (−) 10 ng/ml IL-18. CD16 MFI of CD56^dim^CD16^+^ NK cells was measured after 6 or 18 h **(C)** (*n* = 7–16, data from one to two experiments). Surface expression of CD107a **(D)**, intracellular IFN-γ **(E)**, and CD25 **(F)** was measured on NK cells after 6 or 18 h (*n* = 7–16, data from one to two experiments). Box plots show the 5th to 95th percentile range. Data were analyzed using paired Wilcoxon signed-rank tests [**(C–F)** capped lines] or ANOVA tests for linear trend for trend analysis across increasing cytokine concentrations including Med [**(B)** uncapped lines]. ****p < 0.0001, ***p < 0.001, **p < 0.01, *p < 0.05.

After vaccination, or upon secondary infection, circulating antigen–antibody complexes binding to FcγRIII (CD16) on NK cells can mediate killing of infected cells *via* ADCC. As our data suggest that IL-18, in concert with γ_c_ cytokines, enhances adaptive and innate pathways of NK cell activation, we wanted to test whether ADCC could be augmented by very low levels of NK cell activating cytokines, as would be present at the site of infection (Figure 4).

We found that IL-18, but not IL-12, IL-15, or IL-21, induced rapid (within 6 h) and sustained (persists at 18 h), concentration-dependent downregulation of CD16 expression at the NK cell surface such that cells substantially lost CD16 expression within 6 h in the presence of 10 ng/ml IL-18 (Figures 4A,B; Figure S2 in Supplementary Material). We have previously observed that cross-linking of CD16 with plate-bound anti-CD16 antibody leads to loss of CD16 from the NK cell surface (Goodier, unpublished data), and this is consistent with previous reports of CD16 downregulation following CD16 ligation (28). Here, we observe that the inherent capacity of IL-18 to reduce CD16 expression synergizes with the effects of CD16 cross-linking such that after 6 and 18 h, residual CD16 expression is lower when NK cells are cultured with 10 ng/ml IL-18 plus plate-bound anti-CD16 than when they are cultured with either anti-CD16 or IL-18 alone (Figure 4C). Taken together with data indicating that downregulation of CD16 on CD56^dim^ NK cells in response to either CD16 cross-linking or to very high concentrations of IL-12 (10 ng/ml) plus IL-18 (100 ng/ml) can be blocked with a specific inhibitor specific of the metalloprotease ADAM-17 (28), these data raise the interesting hypothesis that the IL-18 and the CD16 signaling pathways may converge to induce metalloprotease-mediated cleavage of CD16 from the cell surface. We could not find any evidence that CD16 cross-linking affects expression of IL-18Rα (data not shown).

Using cross-linking of cell surface CD16 with plate-bound anti-CD16 antibody as a model of ADCC (28), we next determined the effects of low concentrations of IL-18 on NK cell cytotoxicity [assessed using the CD107a degranulation assay (29, 30)] as well as on CD25 and IFN-γ responses. Despite the very rapid downregulation of CD16 by IL-18 and CD16 cross-linking (Figures 4B,C), we observed that as little as 10 ng/ml IL-18 markedly and very rapidly (within 6 h) augmented NK cell degranulation and IFN-γ production in the presence of anti-CD16 antibody (Figures 4D,E). Furthermore, IL-18 synergized with anti-CD16 to enhance CD25 expression at 18 h (Figure 4F; test for interaction, *p* = 0.005). These data demonstrate that IL-18 can substantially enhance NK cell ADCC responses and also support the idea that IL-18- and anti-CD16-driven downregulation of CD16 expression is a consequence of activation of signaling pathways downstream of CD16.

**Figure 5:**
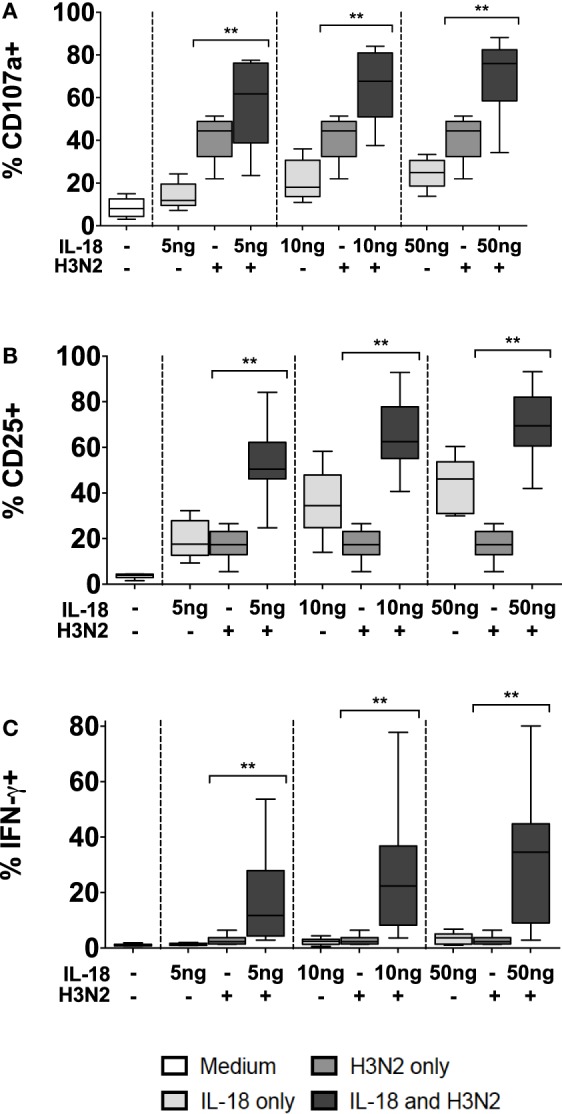
**Antibody and IL-18 synergism in response to H3N2**. PBMCs were stimulated for 18 h *in vitro* Med (medium alone), 5, 10, and 50 ng/ml IL-18 with or without 1 μg/ml inactivated influenza virus H3N2. Responses were measured as the percentage of NK cells expressing surface CD107a **(A)**, surface CD25 **(B)**, or intracellular IFN-γ **(C)** (*n* = 7–8, data from one experiment). Box plots show the 5th to 95th percentile range. Data were analyzed with paired Wilcoxon signed-rank tests. ****p < 0.0001, ***p < 0.001, **p < 0.01, *p < 0.05.

To validate this apparent interaction between IL-18 and CD16, PBMCs were incubated for 18 h with or without whole, inactivated H3N2 influenza virus in the presence of plasma that had previously been shown to contain anti-H3N2 IgG and to mediate ADCC [(6); Goodier et al., submitted]. NK cell degranulation, CD25, and IFN-γ responses to H3N2 immune complexes were compared in the presence or absence of exogenous IL-18 (Figure 5). As reported above, IL-18 alone induced modest increases in CD107a, CD25, and IFN-γ expression. Furthermore, as previously reported for H1N1 virus (6), incubation of NK cells with H3N2 virus and plasma containing anti-H3N2 antibodies induced significant degranulation (CD107a expression) (Figure 5A), upregulation of CD25 (Figure 5B), and production of IFN-γ (Figure 5C). However, as little as 5 ng/ml IL-18 in combination with H3N2 and anti-H3N2 was sufficient to markedly augment CD107a, CD25, and IFN-γ expression; the effects of the combination of IL-18 plus H3N2/anti-H3N2 were additive for CD107a expression, but synergistic for CD25 and IFN-γ (Figures 5A–C, tests for interaction, 5 ng/ml IL-18 and H3N2 for CD107a *p* = 0.269; CD25 *p* = 0.002; and IFN-γ *p* = 0.038).

In summary, therefore, IL-18 synergizes with CD16-mediated signals to augment NK cell ADCC activity and indicates that IL-18 may play an important role in driving NK cell cytotoxicity and cytokine production.

## Discussion

The capacity of NK cells to be activated, within minutes or hours, by very low concentrations of innate cytokines is integral to their role as early responders during infection. Although pathogens may, ultimately, be cleared by components of the adaptive immune system, NK cells and other innate leukocytes are critical for initial containment of infection and orchestration of the subsequent adaptive response (31).

The role of exogenous cytokines in driving NK cell responses has been studied extensively in different contexts, including infection and cancer immunotherapy. However, these *in vitro* experiments have, almost exclusively, been carried out with purified NK cells stimulated with very high concentrations of cytokines that do not reflect the *in vivo* response, may over-ride natural homeostatic mechanisms that regulate the extent and duration of NK cell activation, and ignore interactions with other immune cells and with components of the adaptive immune system. Importantly, few studies have evaluated combinations of more than two cytokines and none have carefully titrated cytokine concentrations within these combinations. Thus, although we have abundant information about which cytokines and other signals can, under certain conditions, activate NK cells, we have a much less clear picture of which signals and which combinations of signals most efficiently activate NK cells in physiologically relevant conditions.

In an attempt to conduct a more physiologically relevant analysis of NK cell–cytokine interactions, we have conducted a systematic analysis of the roles of different cytokines and cytokine combinations in NK cell activation and their interaction with adaptive immune responses. We have demonstrated that NK cells can respond, within hours, to concentrations of cytokines that are orders of magnitude lower than previously appreciated and that NK cells can integrate signals, synergistically, from multiple cytokine receptors and FcγRIII, enabling them to respond quickly and effectively to extremely low concentrations of pro-inflammatory stimuli.

Our study differs from published work not only in the very low concentrations of stimuli used, and the use of multiple cytokines in combination, but also in that we have analyzed NK cell responses within whole PBMCs (containing other lymphocyte populations as well as monocytes, macrophages, and dendritic cells) rather than using purified NK cells. Although the use of purified NK cells removes extraneous signals and allows the effects of precisely controlled cytokine concentrations to be evaluated, this approach negates the potential role of secondary cytokine responses and cell–cell contact-mediated signals, which may potentiate the effect of the test stimulus (1). We fully accept that, in our assays, exogenous cytokines might induce other cells in the PBMC population to express accessory molecules or produce cytokines that augment NK cell responses, and indeed this may explain the rather limited contribution of exogenous IL-12 in our assays, but we argue that this better reflects the *in vivo* situation and the true potential of NK cells. Both tissue-resident NK cells and circulating NK cells that infiltrate inflamed sites will receive co-stimulatory signals from myeloid and lymphoid cells present at the site of infection, and this interplay between different components of the immune system is crucial to rapid containment of infection.

Removing accessory cells from culture abrogates many important cell–cell signals; for example, contact between NK cells and DCs is required for optimal presentation of IL-15 *via* IL-15-IL-15Rα complexes, reducing the minimally effective IL-15 concentration from the nanomolar to picomolar range (32, 33). Similarly, interactions between NK cells and macrophages mediated, for example, by NKG2D and ICAM–LFA-1, are required for NK cell activation in numerous infection models [(34, 35) and reviewed in Ref. (1, 36)]. In the absence of these co-stimulatory signals, responses of isolated NK cells to rather high concentrations of exogenous cytokines represent only a very incomplete picture of their true potential. Moreover, NK cell isolation may, in itself, introduce artifacts. Negative selection with agonistic anti-CD3 antibodies risks leaving behind a residue of highly activated, cytokine-producing T cells that may confound analysis of responses to other cytokines, and positive selection of NK cells requires cross-linking of surface receptors that may positively or negatively affect the subsequent NK cell response. On balance therefore, we suggest that the experiments reported here better reflect physiological conditions during early infection and provide novel insights into NK cell activation in this context. Although circulating NK cells are the only easily accessible population in humans, experiments using tissue-resident NK cells, such as lung-resident NK cells in the context of influenza infection, would also be of interest.

Our data point to IL-18 as the key component of the initial inflammatory response that “primes” NK cells to respond to other cytokines and to FcγRIII/CD16-mediated signals. In accordance with published data (12), we show that 50 ng/ml IL-18 is sufficient to induce CD25 expression on >40% of NK cells within 18 h; however, we extend these data to reveal significant upregulation of CD25 within 6 h at IL-18 concentrations as low as 10 ng/ml. Rapid IL-18-induced upregulation of CD25 explains the synergistic interaction we observed between IL-18 and IL-2; this has been reported previously but only at IL-18 concentrations that are 100-fold (10) higher than the concentration used here. Moreover, our data extend these findings to demonstrate that synergism between IL-18 and IL-2 enhances IFN-γ production (irrespective of the presence of IL-12 or IL-15) and we confirmed the importance of IL-18 in enhancing ADCC responses to influenza virus *via* naturally occurring anti-H3N2 antibodies in normal human plasma. Taken together, these data place IL-18 at the interface of innate and adaptive activation of NK cells.

Our data reveal that γ_c_ cytokines are key partners of IL-18 in this process. IL-2, IL-15, and IL-21 all signal *via* the common γ chain, CD132, while receptors for IL-2 and IL-15 also share the β subunit, CD122 [(21, 37) and reviewed in Ref. (38)]. Although IL-2 and IL-15 are often considered functionally interchangeable as a consequence of their shared STAT5 signaling pathway [reviewed in Ref. (38)], we find that resting NK cells are much more sensitive to IL-15 than to IL-2. This likely reflects the very low levels of expression of the high affinity IL-2Rα (CD25) on resting NK cells; once CD25 is upregulated, IL-2 is not only an extremely potent inducer of NK cell IFN-γ production but also further upregulates its own receptor in an autocrine, positive feedback loop. Importantly, however, although IL-15 and IL-18 both individually and synergistically upregulate CD25 expression, and IL-18 subsequently synergizes with IL-2 to increase IFN-γ production, there is no such synergy between IL-15 and IL-2. Rather, adding IL-2 to any cytokine cocktail-containing IL-15 reduces CD25 expression and has little if any beneficial effect on IFN-γ production. This is consistent with evidence that IL-2 reduces transcription of *IL15RA* (22), thereby limiting further signaling by IL-15, and may represent an important homeostatic mechanism to constrain innately driven NK cell responses once an effective adaptive immune response is underway.

Sequential activation through shared receptor components, initially by an innate cytokine (IL-15) and thence by an adaptive cytokine (IL-2), would provide a mechanism of NK cell activation that is both efficient and self-limiting, and indeed there is evidence suggesting that sequential activation of human NK cells with IL-15 and then with IL-2 potentiates STAT5 expression (12, 21). Moreover, downregulation of IL-15Rα expression by IL-2 (22) may reduce competition between IL-15Rα and IL-2Rα for the β and γ chains, facilitating formation of high affinity IL-2R and thus potentiating IL-2 signaling. Competition between IL-2 and IL-15 may extend to the shared STAT5 pathway and may explain the lack of competition with IL-21 that, although sharing the common γ chain receptor, signals *via* STAT3 (38).

We show that IL-18 also synergizes with IgG/CD16, with as little as 10 ng/ml IL-18 enhancing NK cell degranulation and IFN-γ production within 6 h. IL-18 has previously been reported to enhance IFN-γ production and ADCC following CD16 ligation, albeit only at a 10-fold higher IL-18 concentration than employed here (39). The discrepancy between these two studies may be explained by the much longer (overnight) incubation times used by Srivastava et al. (39) as we found that IL-18/anti-CD16-induced responses were well past their peak after 18 h, possibly because of the very rapid downregulation of CD16 expression by IL-18 that we also observed. Although downregulation of CD16 by IL-18 has been observed previously (15, 39), it was reported only at high IL-18 concentration (100 ng/ml or 1 μg/ml) after periods of 1–5 days and the effect was not fully quantified. The speed with which CD16 is downregulated, the very low concentrations of IL-18 required to induce it, and the synergy with CD16 cross-linking have not previously been appreciated and may, again, represent a homeostatic control mechanism to prevent excessive NK cell cytolytic activity and associated tissue damage.

**Figure 6:**
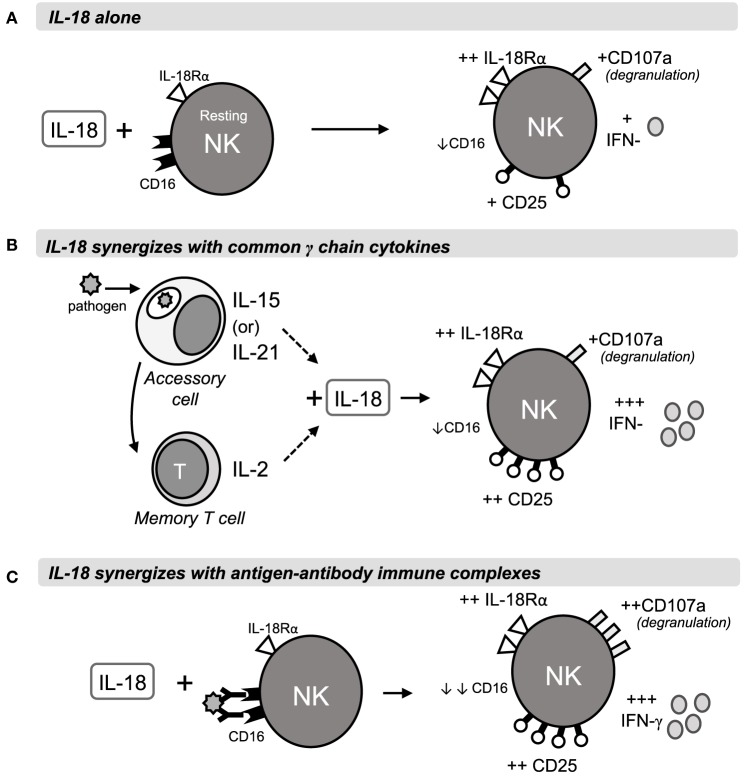
**Rapid IL-18 driven activation of NK cells**. The schematic shows a proposed model of NK cell activation by IL-18 and its synergies with γ_c_ cytokines or antigen-antibody immune complexes during primary or secondary immune responses. Within 6 h of stimulation, IL-18 drives IL-18R upregulation and modest increases in CD25, IFN-γ and degranulation, as well as downregulation of FcγRIII (CD16) **(A)**. In combination with IL-15 or IL-21 from accessory cells, or with T cell-derived IL-2, IL-18 drives much stronger CD25 and IFN-γ responses, measurable at 6 h and peaking at 18 h **(B)**. Antigen-antibody complexes cross-link CD16 and synergise with IL-18 to drive ADCC and IFN-γ production after 6 and 18 h **(C)**.

Taken together, our data lead us to propose the following model of early NK cell activation (Figure 6). At the start of a primary infection, the initial colonizing pathogens induce release of constitutively expressed IL-15, activation of constitutively expressed IL-18 precursor, and secretion of bioactive IL-18, and transcription and translation of *IL15* and *IL18* by dendritic cells and macrophages [reviewed in Ref. (40–42)]. The synergistic interaction of IL-15 and IL-18 rapidly induces NK cells to produce IFN-γ (within 6 h); this response may be further augmented by IL-12 and is sustained (for at least 18 h) by a positive feedback loop in which IL-18 sustains expression of IL-18Rα. At the same time, induced expression of CD25 allows formation of the high affinity IL-2R, priming the NK cells to take part in T cell-mediated adaptive immune responses. Later in infection, or during re-infection, after the differentiation of antigen-specific T helper cells and production of antibodies, IL-18 synergizes with IL-2 and with antibody-antigen complexes to enhance ADCC and IFN-γ production. The immediate availability of IgG antibodies allows ADCC reactions to occur within 5 h (28), and subsequent rapid downregulation of FcγRIII/CD16 by IL-18 and/or CD16 cross-linking brings the reaction to a close, thereby preventing immune pathology. As IL-2 signaling commences, downregulation of IL-15Rα and/or competition for β and γ chain receptor components inhibits further IL-15 signaling. IL-2Rα expression is now sustained by IL-18 and IL-2, further enhancing NK cell sensitivity to IL-2 and, in synergy with IL-12, maximizing IFN-γ production (12).

In summary, therefore, we have shown that IL-18 synergizes with components of both the innate (IL-15, IL-12) and the adaptive immune response (IL-2, antibody) to very rapidly induce antimicrobial NK cell responses (IFN-γ and ADCC). The extremely low concentrations of cytokines that are required for this process, and the speed with which it happens, identify IL-18 as a key “first responder” at the intersection of innate and adaptive immune responses to infection.

## Author Contributions

CN and A-SW designed and carried out the experiments and analysis. MG and ER advised on experimental design and analysis. CN, A-SW, and ER wrote the paper.

## Conflict of Interest Statement

The authors declare that the research was conducted in the absence of any commercial or financial relationships that could be construed as a potential conflict of interest.
