# Supplementary material for: Synergy between Common γ Chain Family Cytokines and IL-18 Potentiates Innate and Adaptive Pathways of NK Cell Activation
Source: Front Immunol. 2016 Mar 22;7:101. doi: 10.3389/fimmu.2016.00101 (PMC4801862; doi:10.3389/fimmu.2016.00101)

Supplementary Material

Synergy between common γ chain family cytokines and IL-18 potentiates innate and adaptive pathways of NK cell activation

Carolyn M. Nielsen*, Asia-Sophia F.M. Wolf*, Martin R. Goodier, Eleanor M. Riley§.

§ **Corresponding author:**
Professor Eleanor M. Riley eleanor.riley@lshtm.ac.uk tel: 020 7927 2706

This supplementary material shows an extended titration of IL-12 and IL-18 in combination, titrations of IFN-α and IL-21, and the effects of various cytokines on IL-12Rβ2.

Supplementary Figure 1. Extended IL-12/IL-18 titrations. PBMC were stimulated with medium alone, or increasing concentrations of IL-12 and/or IL-18 (as labelled on graphs). NK cell surface expression of IFN-γ (**A**) and CD25 (**B**) after 18 hours. *n*= 2, data from one experiment.

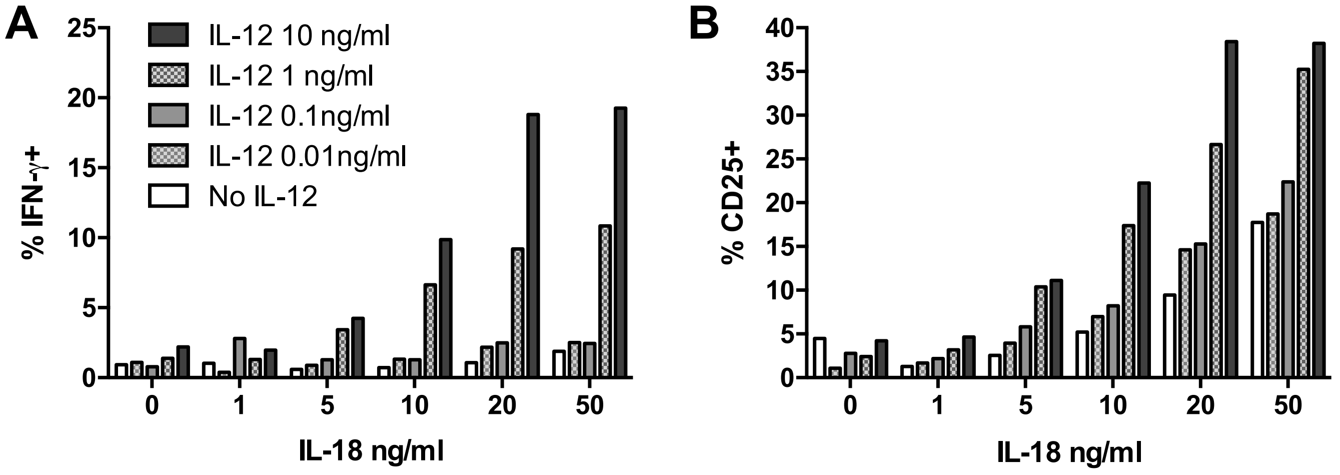

Supplementary Figure 2. IFN-α and IL-21 titrations. PBMC were stimulated with increasing concentrations of IFN-α or IL-21. NK cell surface expression of CD25 was measured in response to Med (medium alone), 10pg, 100pg or 1000pg/ml IFN-α alone, or HCC, a positive control of high concentration IL-12 (5ng/ml) and IL-18 (50ng/ml), at 6 hours **(A)** and 18 hours **(B)**, and to combinations of IFN-α (10pg/ml) and IL-2 (5ng/ml), IL-12 (12.5pg/ml), IL-15 (0.75ng/ml) or IL-18 (10ng/ml) after 18 hours **(C)** (*n*= 8, data from 2 experiments). Likewise, intracellular production of IFN-γ was measured after 18 hours in response to medium alone, IFN-α alone, or HCC **(D)** or in combination with IL-2, IL-12, IL-15 or IL-18 as previously mentioned **(E)** (*n*= 4, data from 1 experiment). Expression of IL-18Rα was also measured at 6 and 18 hours in response to IFN-α titration **(F)** and IL-21 titration **(G)** at the cytokine concentrations indicated on the graphs (*n*= 4, data from 1 experiment). Finally, CD16 MFI of $CD56^{dim}CD16^{+}$ NK cells was measured after 6 or 18 hours **(H)** (*n*= 4, data from 1 experiment).

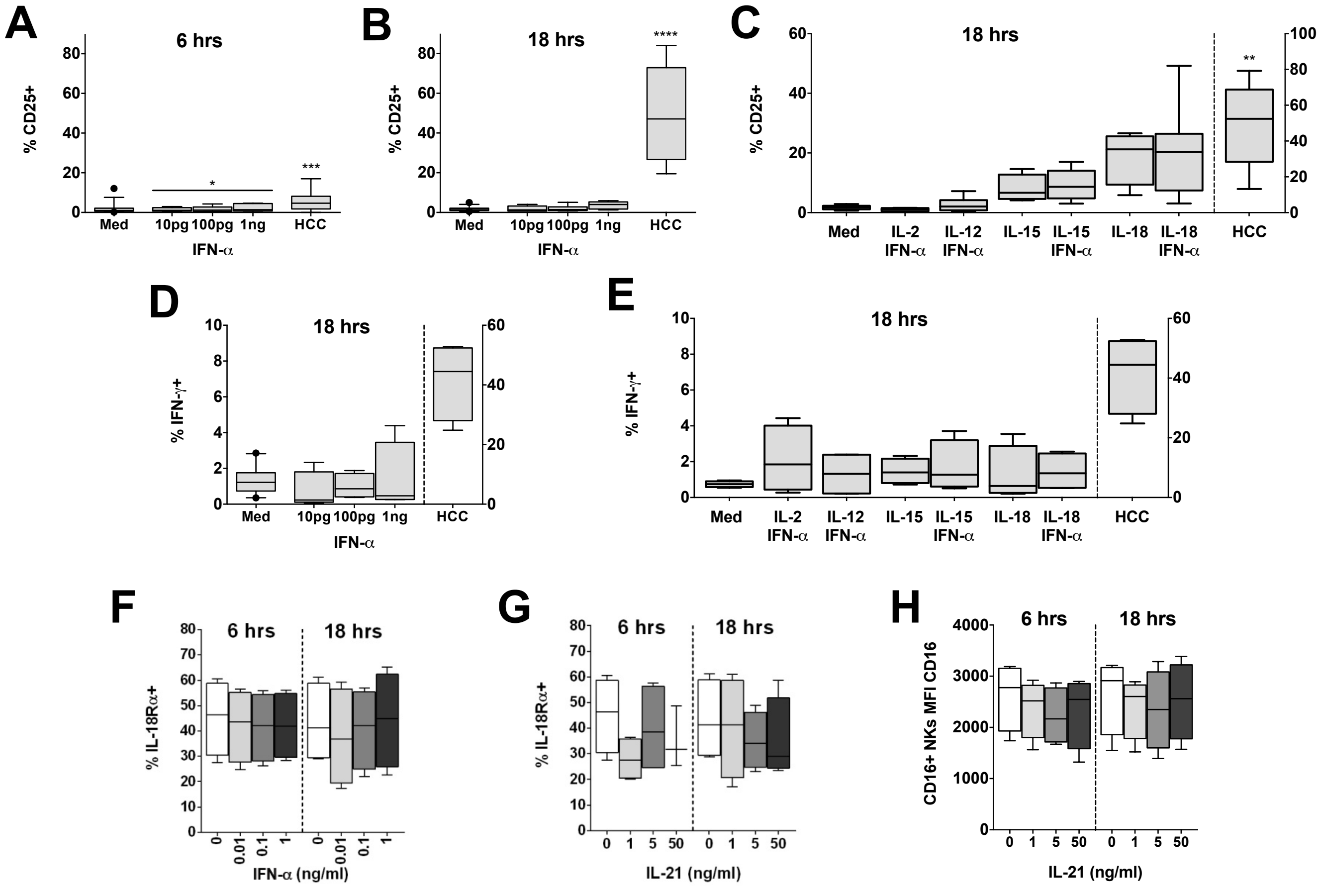

Supplementary Figure 3. Low level IL-12Rβ2 upregulation following IL-15 or IL-18 stimulation. PBMC were stimulated for 6 or 18 hours *in vitro* and changes in NK cell surface expression of IL-12Rβ2 was measured in response to Med (medium alone), IL-2, IL-12, IL-15, or IL-18. Representative flow cytometry plots show gating of $CD3^{-}CD56^{+}$ NK cells and surface expression of IL-12Rβ2 on unstimulated cells **(A)**. IL-12Rβ2 expression on NK cells was measured after stimulation with Med, IL-2, IL-12, IL-15, IL-18 (concentrations in ng/ml as labelled) or HCC after 6 hours **(B)** (*n*= 7-16, data from 1-3 experiments) or 18 hours **(C)** (*n*= 8-18, data from 2-3 experiments), with mean fluorescence intensity (MFI) of IL-12Rβ2 expression on NK cells for the same experiments at 6 hours **(D)** and 18 hours **(E)**. NB: IL-2 titrations were performed using a different batch of anti-IL-12Rβ2-PerCP/Cy5.5 conjugated antibody. Data were analysed using paired Wilcoxon signed-rank tests (B-C: no lines; compared to Med) or ANOVA tests for linear trend for trend analysis across increasing cytokine concentrations including Med (B-C, uncapped lines). ***p<0.001, **p<0.01, *p<0.05.

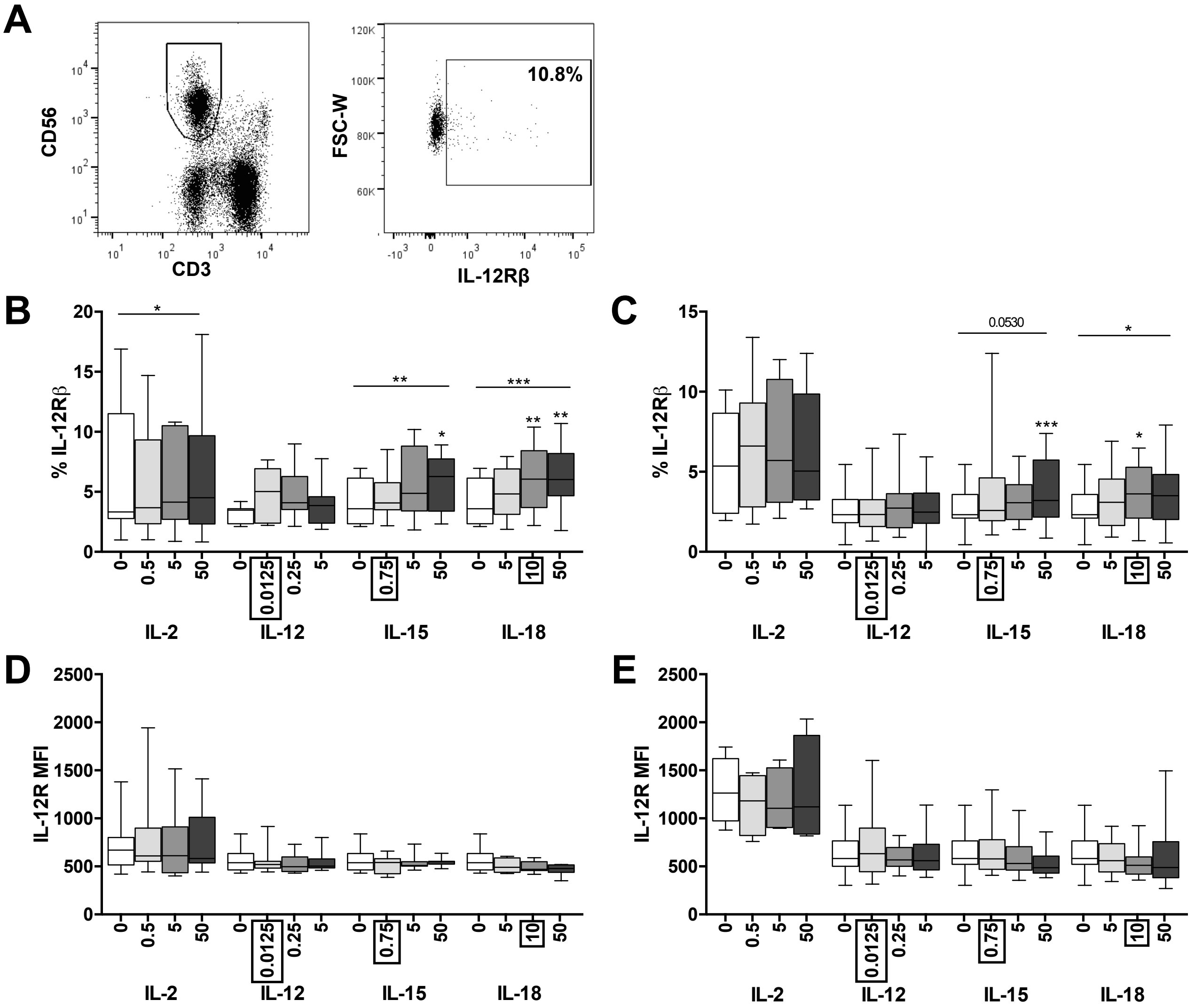

Supplement: Supplementary file 1 [file Presentation_1.pdf]
